# Progressive compressive sensing of large images with multiscale deep learning reconstruction

**DOI:** 10.1038/s41598-022-11401-7

**Published:** 2022-05-04

**Authors:** Vladislav Kravets, Adrian Stern

**Affiliations:** Department of Electro-Optics and Photonics Engineering, School of Electrical and Computer Engineering, Ben-Gurion University of the Negev, P.O.B. 653, 8410501 Beer-Sheva, Israel

**Keywords:** Optical techniques, Imaging and sensing

## Abstract

Compressive sensing (CS) is a sub-Nyquist sampling framework that has been employed to improve the performance of numerous imaging applications during the last 15 years. Yet, its application for large and high-resolution imaging remains challenging in terms of the computation and acquisition effort involved. Often, low-resolution imaging is sufficient for most of the considered tasks and only a fraction of cases demand high resolution, but the problem is that the user does not know in advance when high-resolution acquisition is required. To address this, we propose a multiscale progressive CS method for the high-resolution imaging. The progressive sampling refines the resolution of the image, while incorporating the already sampled low-resolution information, making the process highly efficient. Moreover, the multiscale property of the progressively sensed samples is capitalized for a fast, deep learning (DL) reconstruction, otherwise infeasible due to practical limitations of training on high-resolution images. The progressive CS and the multiscale reconstruction method are analyzed numerically and demonstrated experimentally with a single pixel camera imaging system. We demonstrate 4-megapixel size progressive compressive imaging with about half the overall number of samples, more than an order of magnitude faster reconstruction, and improved reconstruction quality compared to alternative conventional CS approaches.

## Introduction

**Figure 1:**
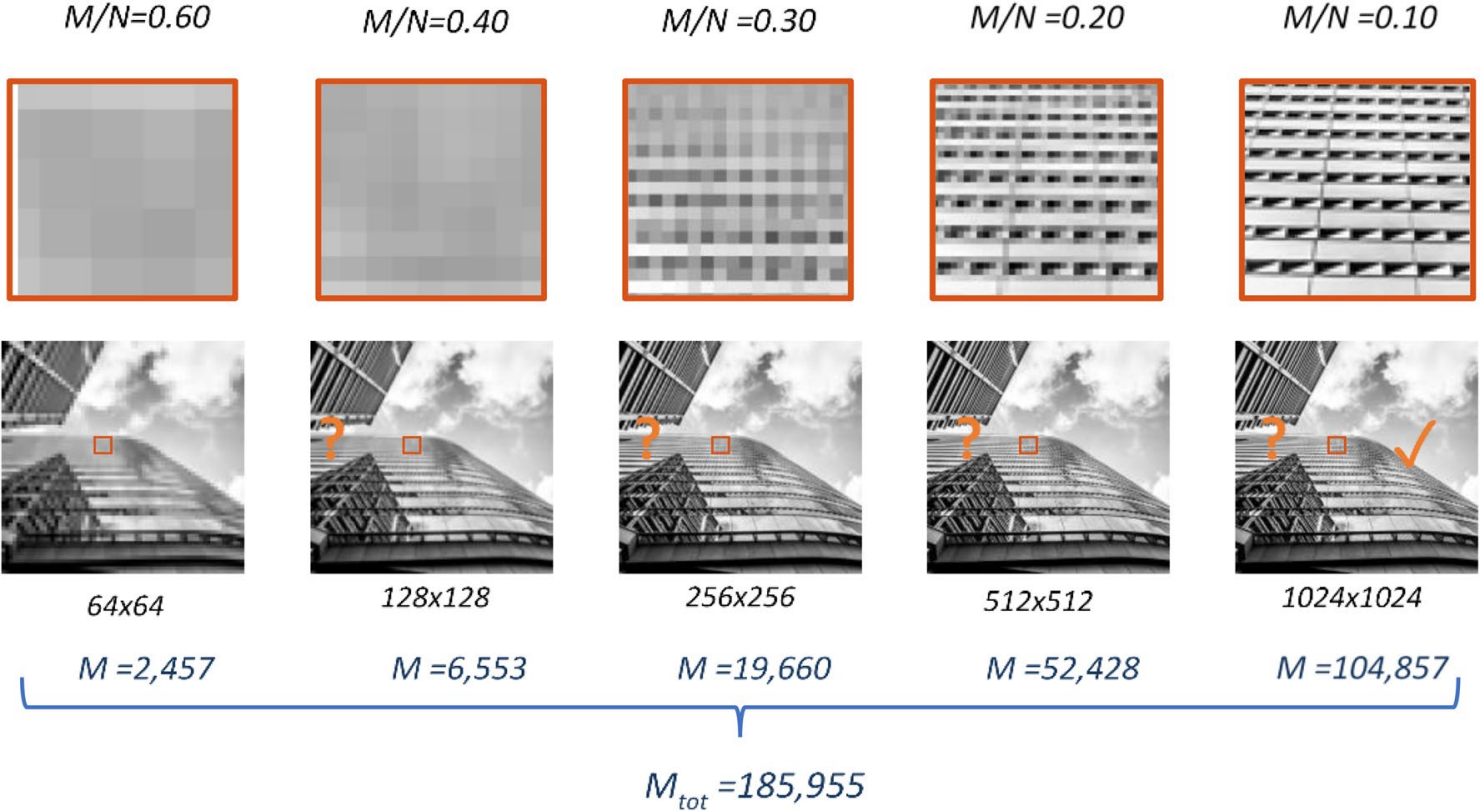
Illustration of CS reconstruction of an image of a multi-story building at different resolutions. The images are sorted from low resolution to high, from left to right. *M* denotes the number of samples that must be taken to sample at the compression ratio *M*/*N*, specified in the top row. The square area outlined in red is enlarged in the row above each image to illustrate the increase of fidelity at each resolution.

Compressive sensing (CS)^1–3^ is a sensing technique that allows sub-Nyquist sampling rates of natural signals. Therefore, CS was found to be very useful for imaging systems that require large acquisition efforts, such as capturing large images, multidimensional images, or when exhaustive scanning is required^1^. A principal property of CS theory is that it provides the user with guidelines about the number of compressive samples needed to reconstruct an image of a given size, *N*. However, a common problem CS practitioners encounter is that it is often difficult to predict the desirable quality of the reconstructed image before its capture. If an over-optimistic *N* is assumed when designing the acquisition step, the resulting reconstruction may not exhibit sufficient detail. Then, the sampling process might be repeated from scratch, but with finer detailed compressive patterns under the assumption of a larger *N*. Figure 1 demonstrates such a scenario. In Fig. 1 iteratively refined compressive imaging^4^ of a multi-story building is illustrated. Let us assume, for example, that our task is to count the number of floors in buildings in a city. For low-rise buildings, usually a 64 by 64 resolution image would be sufficient. Therefore, there is no need to always sample with the highest resolution. However, when an image of a tall building is taken, we may find the upper floors indistinguishable. Therefore, we would change the CS patterns to match the increased resolution and sample the image again, repeating this process until a satisfactory image is obtained. There are two main problems with this practice. First, the acquisition process is extremely inefficient because the samples taken in each refinement step are agnostic to the information captured in the previous trial. Second, a large reconstruction effort is required, because it is extremely time consuming to repeatedly reconstruct the image using conventional iterative algorithms.

In this paper, we propose a new CS procedure that prescribes which set of samples should be added to the previously captured ones in order to improve the resolution by the desired amount. For this scenario, we developed a deep learning (DL) approach for fast and efficient image reconstruction.

With our approach, we sample the image progressively, capturing only the necessary additional finer scale sensing patterns, while still using the previously taken coarse-detail information. We increase the resolution while decreasing the compression ratio, as the higher resolution images can be better compressed. In the example in Fig. 1, if the resolution is still not sufficient to count the number of stories, we increase the resolution again, while decreasing the compression ratio.

This scanning scenario is especially relevant for systems such as a light detection and ranging (LIDAR) system. For LIDAR systems designed for very long-range 3D scanning (more than 10 km) it is often required to improve the resolution in order to detect certain hidden objects. Therefore, instead of scanning the scene from the start to fit a higher resolution, it is much more efficient to add patterns while still using the previous information, and thus to reduce the sampling time.

Notice in Fig. 1 that the number of compressive samples, *M*, necessary to sample the image at 1024 by 1024 resolution is around 10^5^ samples, which is almost half the total number of samples taken, denoted by *M*_*tot*_. Therefore, if we take the compressive samples from scratch at each resolution, the total number of samples would have been almost double the number taken in the highest resolution image from the start. Instead, with our proposed method, we sample the image progressively, and only add the number of samples needed to achieve the higher resolution, reaching the same number of samples as if we had sampled at the highest resolution from the start.

An important property of the proposed method is that it allows us to compressively sample large images. In general, compressive sampling of high-resolution images is challenging, during both the sampling stage and the reconstruction stage. In order to sample high-resolution images, the sensing patterns have to be generated iteratively on the fly, because storing pre-set patterns in the memory is not feasible in practice. For example, to compress the 1024 by 1024 image in Fig. 1 by a ratio of 10:1, using random binary patterns^5^, a sensing matrix with more than $1{0}^{11}$ entries is necessary, which, if stored in double precision, requires almost 1 Tb of computer RAM. To solve this, the Hadamard basis^6,7^ can be used; this has a fast generative formula, thus preventing the need to store the whole set of patterns in the computer memory. Here, we combine the multiscale property of the Hadamard basis^8^ with the multiscale CS sampling concept^9^ to selectively use the set of Hadamard samples required for each resolution refinement. To facilitate a systematic process that is scalable for large images, we developed a simple method for choosing the multiscale samples needed to capture the 2D compressed images, and we proved the multiscale property in two dimensions (Supplement A).

Another important property of the proposed approach is that it reduces the reconstruction time dramatically compared to classical CS iterative algorithms. This is achieved by a DL reconstruction algorithm that we developed for our scenario. Recently, several DL methods have been applied in the field of CS to reduce the reconstruction time over iterative minimization methods^10–17^, and even real-time CS has been introduced for low-resolution images^16^. Most of the methods work by reconstructing the compressive samples with a fully connected first layer that maps the samples to the image. These methods work fairly well for low-resolution (e.g., 128 by 128 pixels), highly compressed images, or sensing and reconstruction in patches. However, the existing methods are not designed to be used for both high-resolution compressed images and progressive change in resolution. In principle, DL methods developed for small images can be applied to large images by dividing the compressive image sampling into small, compressed patches. However, such a solution is suboptimal in the CS sense, because the number of compressive samples *M* needed to reconstruct the signal of length *N*, is proportional to $\mathit{log}\left(N\right)$^1–3^, therefore the compressibility increases with the signal length. Dividing the image into small parts significantly reduces the compressibility. On the other hand, if we wish to reconstruct the full-resolution images, *without employing patch-wise processing*, severe limitations on the computer memory can arise during the training of the network because of the size of the high-resolution images. We solve this by reconstructing the image from coarse to fine resolution, according to the progressive acquisition process. This enables training on small *patches* that reconstruct only high-frequency details per each scale of the compressed image, while the low-frequency details are inherited from the previous, lower scales. Thus, our approach offers the advantage of sampling the *complete field of view* of the image by the spatially multiplexed Hadamard samples, as well as the advantage of DL reconstruction which is not limited to patches.

We analyze our progressive CS method using numerical simulations and test it experimentally with a Single Pixel Camera (SPC) system. The SPC is very useful for applications such as infra-red imaging, microscopy, ultrasonic imaging, 3D LIDAR imaging, hyperspectral imaging and many more^1,18–25^. We demonstrate around × 30 improvement in reconstruction time and around 2.2 dB improvement in Peak Signal to Noise Ratio (PSNR) over the iterative reconstruction method in simulation studies on 512 by 512 test images, as well as a × 40 improvement in reconstruction time and around 1.8 dB improvement in PSNR on a 2048 by 2048 exemplary test image. To the best of our knowledge, this is the first time a full 4-megapixel compressive image has been reconstructed using the DL method.

## Results

### Experimental results

**Figure 2:**
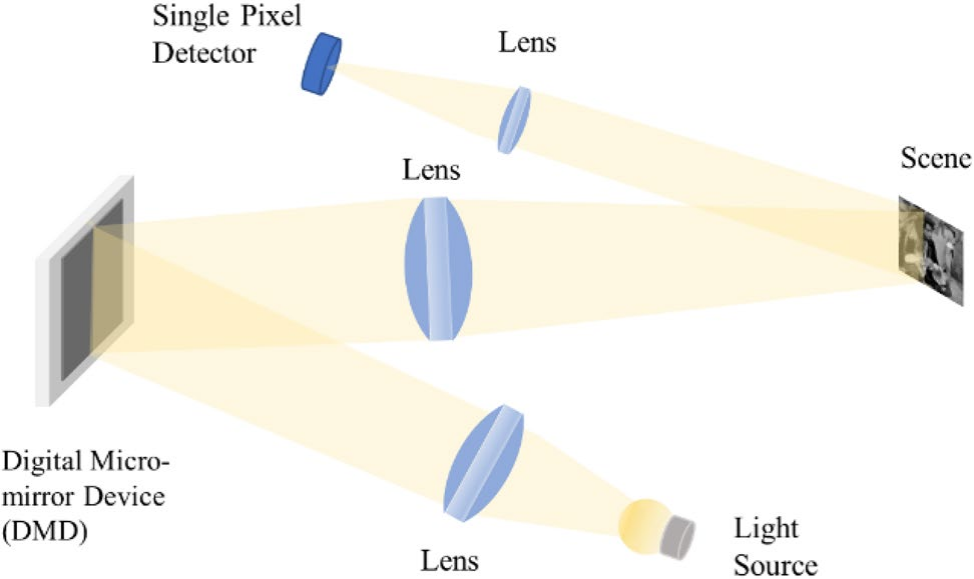
The SPC imaging setup. Halogen light is projected onto the DMD, from which structured light patterns are projected on the scene. A separate single-pixel detector collects the projected light from the scene.

**Figure 3:**
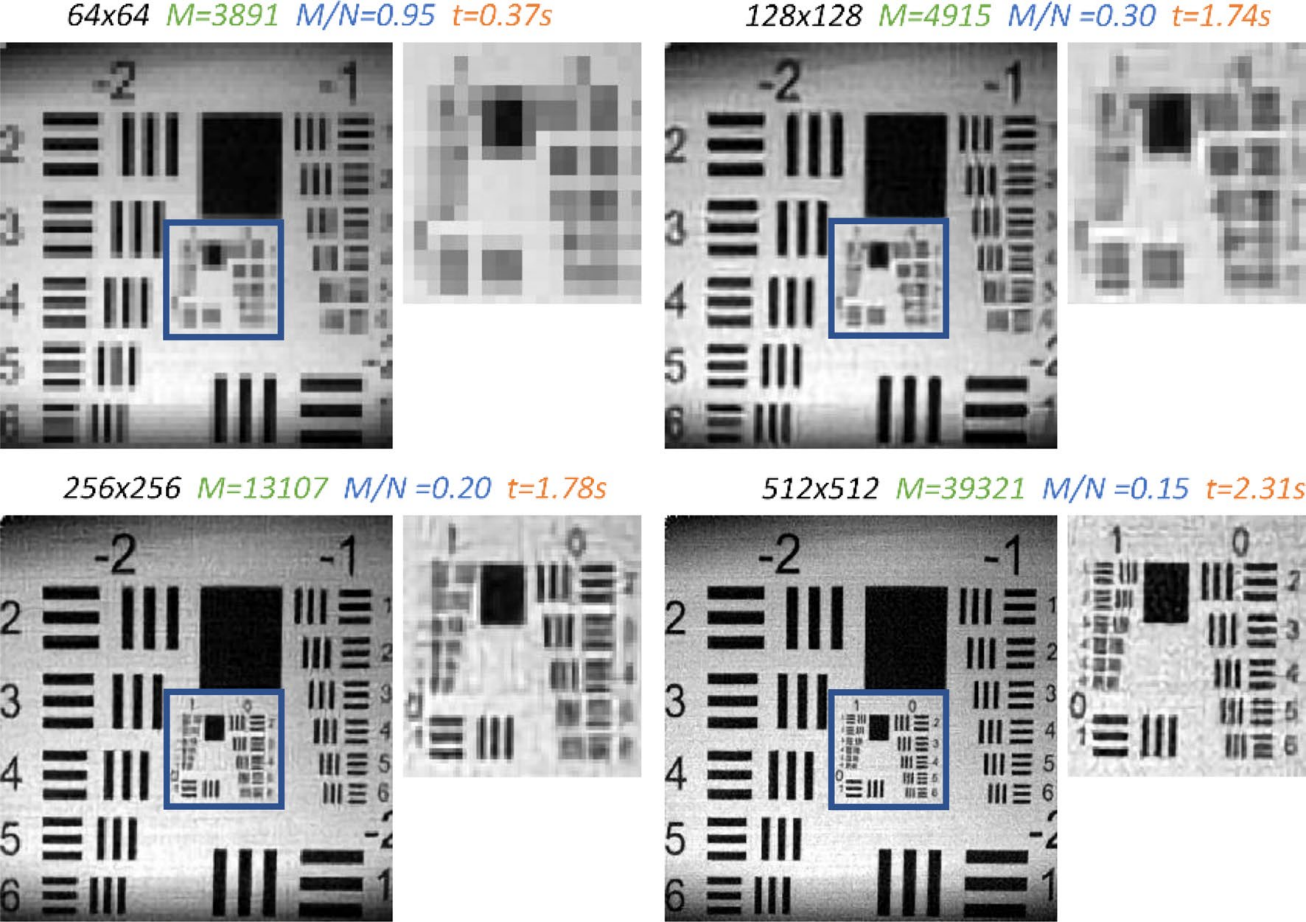
Reconstructions of progressive CS of a USFA MTF target taken by an SPC system in this figure. The number of samples taken at each stage is denoted by *M* in green, the compression is denoted by *M/N* in blue, and the reconstruction time is denoted by *t* in orange.

In Fig. 3 we show progressive CS of the USFA MTF chart taken by an SPC system (see Fig. 2)^18^. The USFA MTF target was taken by progressive compressive samples (see Progressive compressive sampling in the “Methods” section), where compressed Hadamard samples were added at each stage in order to improve the resolution. The reconstruction was performed with the proposed Compressive Multi-Scale network (CMSnet) (see Supplement B). Additional experiment examples can be found in Supplement C.

As it can be seen in Fig. 3, by progressively sensing, the resolution is improved, and therefore more lines at high frequencies can be distinguished at each sampling stage. During the first step of progressive sampling, a coarse image of the size of 64 by 64 pixels is obtained. The user can readily identify that the subject is the USFA MTF target. However, once the user identified the subject, he/she may now wish to identify a higher-resolution group. To accomplish the task, the user may gradually increase the resolution by adding more compressive Hadamard samples chosen to capture only the next resolution level, without restarting the sampling process. Once the user reached the next resolution, if the pair of lines is still indistinguishable, the user can add more Hadamard samples until the lines can be clearly seen. The proposed fast convolutional neural network (CNN) reconstruction approach is especially important in this case, as the conventional iterative approach takes much more time (ranging from one to hundreds of seconds) at each stage.

### Simulations results

**Figure 4:**
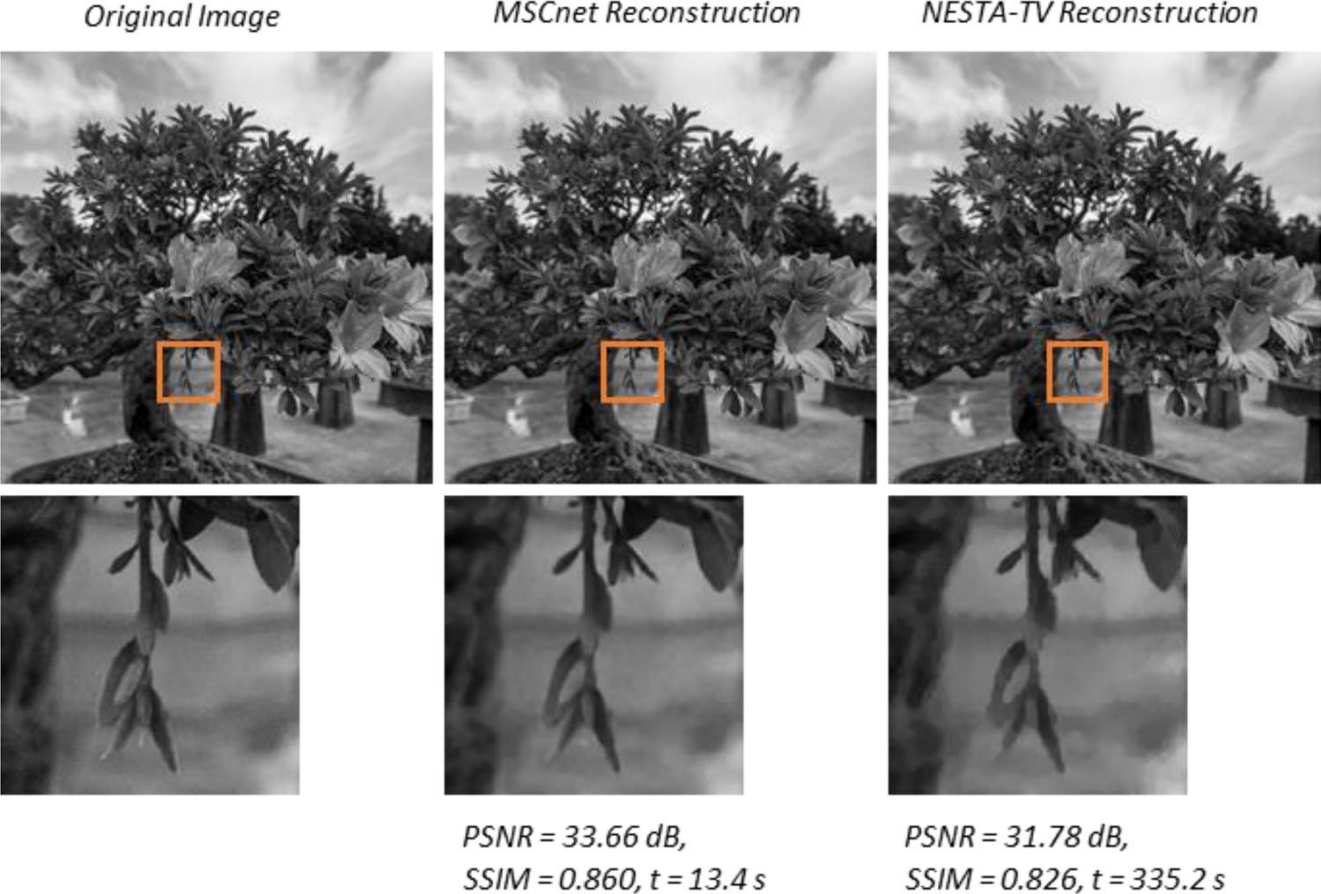
Comparison between a 2048 by 2048 image (top left) and the image reconstruction out of *M/N* = 0.03 compressive samples with the proposed CMSnet (top center) and the iterative NESTA-TV (top right).

In Fig. 4 we compare the proposed CMSnet applied on a progressively sensed large image to an iterative CS reconstruction algorithm that solves a TV minimization problem using the NESTA^26^ solver. Both the proposed DL reconstruction approach and the iterative NESTA-TV algorithm were performed on the same compressive samples. Following the multiscale property of the Hadamard matrix derived in Supplement A, the final Hadamard matrix includes the Hadamard patterns from the lower resolutions. Therefore, we can combine all the previous low-resolution samples into one vector as if they were sampled by a single high order Hadamard matrix. Those samples were used in both the proposed DL and the iterative NESTA-TV reconstruction methods.

We show a reconstruction of a large 2048 by 2048 image of a bonsai tree, in order to demonstrate the ability of the proposed CS method to reconstruct large images. To sample the 2048 by 2048 image we took only 3% of the full Hadamard set. To the best of our knowledge, there is no alternative DL method for direct reconstruction of large CS images sampled with the Hadamard basis, and therefore, we compare our results with the conventional CS iterative reconstruction approach. As can be seen in the comparison in Fig. 4, the CMSnet reconstruction method offers a much more visually pleasing image in comparison to the iterative TV-based CS reconstruction method. This qualitative improvement is manifested in the common quantitative metrics: the proposed CNN method surpasses the iterative approach in PSNR by over ~ 1.8 dB, and in Structural Similarity Index (SSIM) by over ~ 0.03*.* Moreover, CMSnet runs around eighteen times faster than the iterative approach.

In Table 1 we present further comparison between the proposed CMSnet and the iterative NESTA-TV approach for reconstructing the compressive measurements. We compare the methods in terms of PSNR, SSIM and reconstruction time over a sample set of images. The test images that we used in our comparison were Lena, Peppers, Man, Boat, Barbara and Baboon at a 512 by 512 pixels resolution. More comparative results, using other DL methods^10,27–30^, as well as adaptive sensing methods^31,32^, can be found in Supplement B.

**Table 1:** Average PSNR, SSIM and reconstruction time at various compression rates.

Reconstruction	Rate 5%	Rate 10%	Rate 15%	Rate 20%	Rate 25%	Rate 30%
**PSNR**						
CMSnet	**27.16 dB**	**28.95 dB**	**30.90 dB**	**31.47 dB**	**32.64 dB**	**33.55 dB**
NESTA	25.71 dB	27.34 dB	28.43 dB	29.23 dB	29.89 dB	30.49 dB
**SSIM**						
CMSnet	**0.7**7	**0.81**	**0.85**	**0.8**8	**0.90**	**0.9**2
NESTA	0.67	0.74	0.78	0.81	0.84	0.85
**Time**						
CMSnet	**0.309 s**	**0.305 s**	**0.311 s**	**0.304 s**	**0.315 s**	**0.310 s**
NESTA	17.78 s	14.38 s	12.07 s	10.92 s	11.63 s	12.72 s

Significant values are in bold.

For a fair comparison, the reconstruction with the proposed CMSNet and the iterative NESTA methods was performed on the same exact set of compressive samples. In the comparison, we can see that the proposed DL CMSnet approach beats the iterative NESTA-TV minimization approach in PSNR by ~ 1–3 dB, and in SSIM by over ~ 0.08, and it runs around 40 times faster on the 512 by 512 images. We ran our MATLAB simulations on an i7, 32 Gb computer with a GTX1070 GPU.

## Discussion

In this paper, we introduced a progressive CS approach that exploits the multiscale property of the Hadamard matrix, and we developed an appropriate reconstruction procedure. In order to progressively improve the image resolution, additional samples taken at variable densities are added at each stage, until the desirable resolution is reached. A central property of our method is that we use all the previous samples for the reconstruction of the new image resolution, and we add only the set of samples that is needed to capture the next higher-resolution scale.

We note that the proposed progressive sampling method was designed for the sampling of static objects (such as 3D LIDAR surveillance imagers) with a human operator in the loop. This scenario is especially relevant for 3D LIDAR surveillance imagers, and for use in construction, surveying or military applications. While it is possible to use the proposed method on moving objects, future research should explore this further, including examining the stopping criteria method for the progressive sampling and problems associated with objects moving during the sampling.

To improve the reconstruction time and the quality of the reconstructed image, we developed CMSnet, a fast CNN for the reconstruction of compressed images taken with the Hadamard basis. It is important to emphasize that by using a multiscale convolutional approach without fully connected layers, the compressively sampled images are reconstructed at high resolutions that otherwise would be impractical.

In this work, we used the Hadamard basis for the CS. The Hadamard basis has a recursive generative formula, which helps to avoid saving the set of the sensing patterns in the memory, which could otherwise be too large for computer RAM storage. Another advantage to the Hadamard transform is its multiscale property, which was previously^8^ employed by looking for the multiscale patterns that match the lower resolution. However, for very high resolution this process might be very tedious. In this paper (Supplement A) we present a fast and easy method for choosing the multiscale patterns of the 2D Hadamard transform, helping us to work with higher resolution images. To the best of our knowledge, this is the first time that an easy and fast method for choosing the multiscale patterns of the Hadamard matrix is demonstrated.

It is important to note that our CS method is unique in the sense that the *sensing* is implemented on the entire field of view of the image, thus exploiting the compressibility of the entire image, while simultaneously, the *reconstruction* of the image is implemented in patches, thus making the learning and reconstruction more efficient in terms of the computer memory. Additionally, by working with small patches, *the same network can reconstruct images of any size* as we do not have to train a separate network to reconstruct each resolution.

Additionally, we demonstrated a significant advantage in the reconstruction times over the iterative reconstruction approach, while improving the reconstruction quality. We wish to note that the reconstruction quality can be further improved by using a more advanced interpolation method than the Bicubic interpolation as the first approximation of the image. We also note that the reconstruction time can be further shortened at the expense of the image quality by changing the number of times that CMSnet is run at the second step of the reconstruction stage.

To prove the concept on real data, we presented an implementation of the proposed sensing and reconstruction scheme on an SPC imaging system. Our approach allowed us to progressively sample and reconstruct the image while increasing the resolution, without the need to take compressive samples from the beginning. This method can be applied to any of the numerous imaging techniques that use an SPC approach, such as LIDAR imaging^20,33,34^, IR imaging, hyperspectral and ultraspectral imaging^1,18,19,35–38^, polarization imaging^1,19^, three-dimensional imaging, and others. The method could be useful for surveillance tasks, where low-resolution images are taken to detect the existence of a target, and only after the detection, refined sampling is performed for identification.

## Methods

### The sensing scheme

#### Compressive sensing (CS)

CS theory is based on the prerequisite assumption that the signal we wish to sample is sparse or has a sparse representation, meaning that the signal has a small number of non-zero values in some representation. Most humanly intelligible images possess the sparsity property. Another prerequisite is that the measurement matrix should obey certain properties [e.g., the Restrictive Isometry Property (RIP)]^1,3,39^.

The CS sensing scheme can be described by an undetermined linear system of equations:

1 $$ {\mathbf{g}} = {\Phi }{\mathbf{f}} $$

where ${\Phi}\in {\mathbb{R}}^{M\times N}$ is the sensing matrix with *M* < *N*, ${\text{f}}\in {\mathbb{R}}^{N}$ is the signal vector and ${\text{g}}\in {\mathbb{R}}^{M}$ is the measurements vector.

The Hadamard matrix^6^ is a common choice in conventional sensing where the system is realized with a binary sensing mask^7^. In this case, it has been proven that the Hadamard matrix is an optimal matrix because it achieves the Hoteling’s minimum estimation variance^7^. Other useful properties of Hadamard sensing are the availability of the fast Hadamard transform^40,41^, and the fact that the matrix elements do not have to be stored in a memory.

In this paper, we employ the variable density CS concept^42–44^ with the Hadamard matrix^6^, in conjuncture with the multiscale property of the Paley ordered Hadamard matrix, introduced in the next section.

#### The multiscale sensing matrix

We employ the Hadamard matrix as the sensing matrix **Φ** in (1) for CS of the signal. The Hadamard matrix is a self-adjoint, orthogonal matrix. There are various forms of the Hadamard transform for CS^45,46^. Here, we use the Paley ordered Hadamard matrix, which is defined recursively by^6^

2 $$ {\mathbf{R}}_{{\text{n}}} = \frac{{1}}{{\sqrt {2} }}\left( {\begin{array}{*{20}c} {{\mathbf{R}}_{{{\text{n}} - {1}}} \otimes \left( {\begin{array}{*{20}c} {1} & {1} \\ \end{array} } \right)} \\ {{\mathbf{R}}_{{{\text{n}} - {1}}} \otimes \left( {\begin{array}{*{20}c} {1} & { - {1}} \\ \end{array} } \right)} \\ \end{array} } \right) $$

where the matrix **R**_*n*_ is unitary, ${\text{R}}_{0}=1$ and $\otimes$ is the Kronecker product.

The Hadamard matrix has been shown to have a nesting-dolls-like property, where, if ordered, it can retain information of increasing resolutions about the image^8^. This property of the Hadamard basis indicates that it has a certain multiscale behavior. Here, we explore theoretically this multiscale property and provide a useful formulation for its efficient application for CS (Supplement A). This provides us with a method to choose directly the necessary multiscale patterns, as opposed to previous methods^8,9^ that found the patterns manually by comparing the patterns of the high-resolution Hadamard matrix with the lower one.

An important advantage of the multiscale sampling scheme is its ability to reconstruct the sampled image at any chosen scale. This can be utilized, for example, for fast, low-resolution reconstruction that can serve as a viewfinder of real-time images or videos.

As we demonstrate in Supplement A, if we separate the 2D Hadamard transform **G** $\in {\mathbb{R}}^{{2}^{n}{\times 2}^{n}}$ of the image **F** $\in {\mathbb{R}}^{{2}^{n}{\times 2}^{n}}$ into four quadrants, the upper left quadrant **U** $\in {\mathbb{R}}^{{2}^{n-1}{\times 2}^{n-1}}$ is the Hadamard transform of the downscaled (lower-resolution) image ${\text{A}}_{\text{n-1}}\in {\mathbb{R}}^{{2}^{n-1}{\times 2}^{n-1}}$:

3 $$ {\mathbf{G}} = \left( {\begin{array}{*{20}l} {\mathbf{U}} \hfill & \cdots \hfill \\ \vdots \hfill & \ddots \hfill \\ \end{array} } \right) = {\mathbf{R}}_{{\text{n}}} {\mathbf{FR}}_{{\text{n}}} = \left( {\begin{array}{*{20}l} {\frac{{1}}{{2}}{\mathbf{R}}_{{{\text{n}} - {1}}} {\mathbf{A}}_{{{\text{n}} - 1}} {\mathbf{R}}_{{{\text{n}} - {1}}} } \hfill & \cdots \hfill \\ \vdots \hfill & \ddots \hfill \\ \end{array} } \right) $$

**Figure 5:**
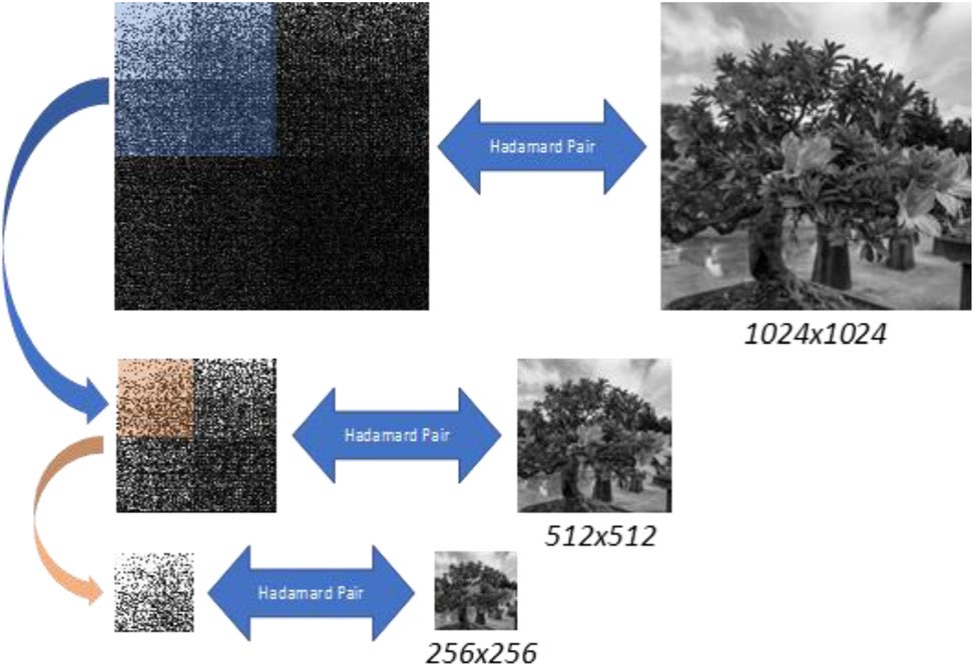
Illustration of the multiscale property of the 2D Hadamard transform (left) and the image (right). The upper left quadrant of the 2D Hadamard transform of an image (in blue) is a Hadamard transform of a lower resolution version of the image. Therefore, we can take the Hadamard samples highlighted in blue and reconstruct from them the 512 by 512 image. In turn, the upper left quadrant of the quadrant (in orange) is also a Hadamard transform of an even lower resolution image. Now, if we take the Hadamard samples highlighted in orange (or a quarter of the samples highlighted in blue) we can reconstruct the 256 by 256 image, directly from the Hadamard samples.

Equation (3) shows that the upper left quadrant **U** of the 2D Paley ordered Hadamard transform of an image is a Hadamard transform of a lower-resolution image **A**. The implication of this property is that, in turn, the upper left quadrant of **U** is a 2D Hadamard transform of a lower resolution of **A**. This *multiscale* property is illustrated in Fig. 5.

#### Progressive compressive sampling

Progressive CS^4^ allows for sampling and reconstruction of the image while gradually improving the resolution by simply adding more compressive samples, without the need to sample and reconstruct the image from the start at each stage.

**Figure 6:**
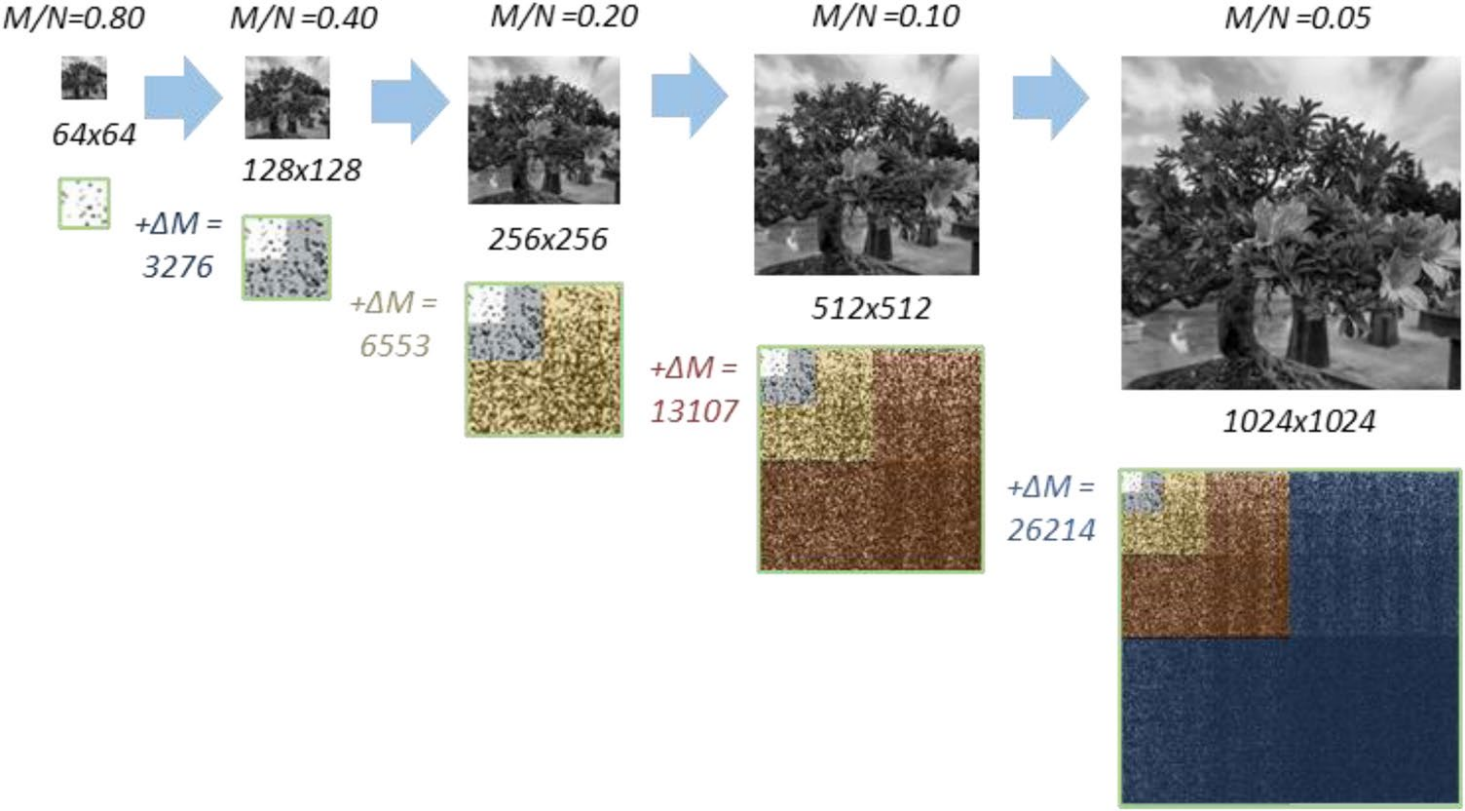
The progressive *sampling* process. The image and its 2D Paley Ordered Hadamard samples below it, are illustrated in sequential order. The first stage starts at a low-resolution image. To increase the resolution, an appropriate additional set of *ΔM* compressive samples, (highlighted in color) is added to the 2D Hadamard transform of the image. The Hadamard samples ordered in 2D are illustrated below the multiscale images.

Progressive CS is performed in stages, as illustrated in Fig. 6. At first, a low-resolution image (e.g., 64 by 64) is taken by the full Hadamard matrix. If the resulting image quality is unsatisfactory, more compressive variable density Hadamard samples^43^ can be added to increase the image resolution. The additional variable density samples are chosen according to the next higher order Hadamard patterns (see Fig. 5). The additional set of Hadamard samples is taken to efficiently improve the resolution, respective to the next higher scale. Only the samples that capture the desired resolution bands are taken; no samples are wasted to capture image details outside the desired resolution bands. The compression at that resolution can also be increased according to user demand. This progressive sampling approach can be repeated iteratively, without the need to start the sampling process from scratch.

### Reconstruction method

**Figure 7:**
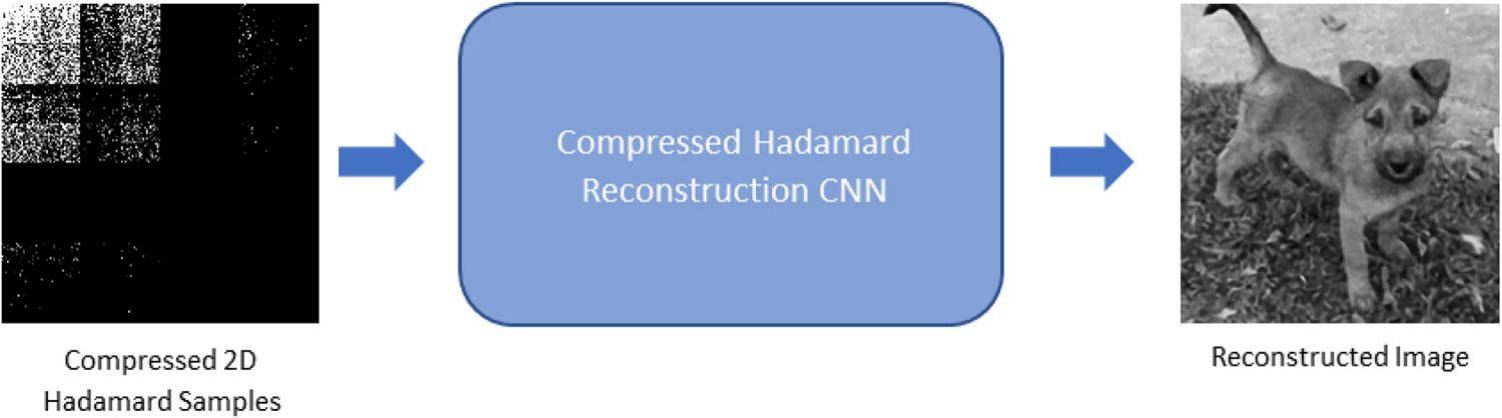
General compressed sensing reconstruction scheme by the means of DL.

We propose a DL-based CNN algorithm to reconstruct the multiscale compressive samples (See Supplement B). One straightforward approach would be to train a CNN to reconstruct the image directly out of the transpose of the Hadamard samples (see Fig. 7).

**Figure 8:**
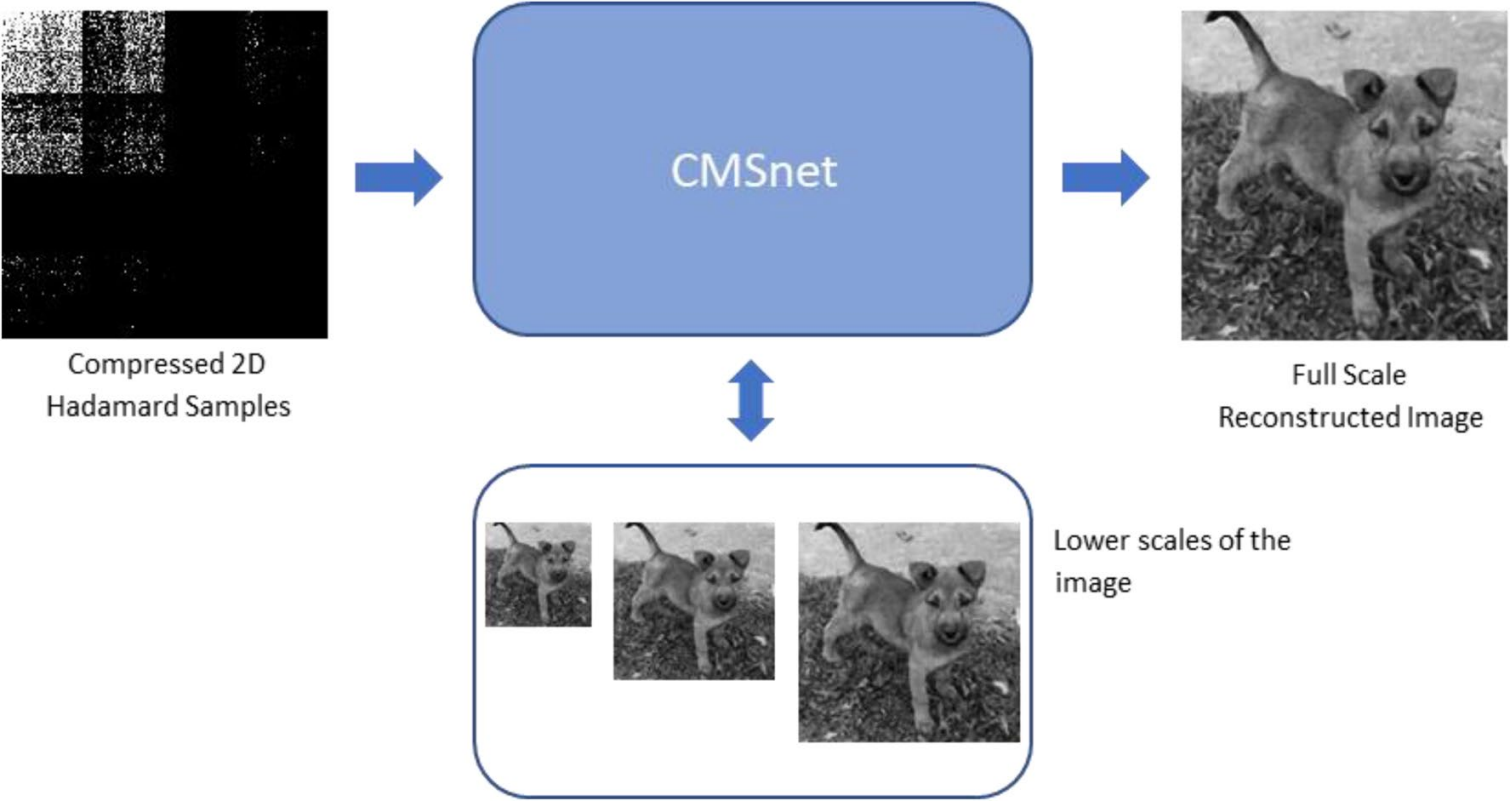
Proposed multiscale compressive sensing reconstruction scheme. The method utilizes the multiscale information that was gathered during the sampling process to improve the reconstruction quality of the image.

This kind of approach is fast and efficient. However, the reconstruction quality can be further improved by using the multiscale property of the Hadamard basis. The proposed progressive sampling method already provides us with multiscale information about the image during the sampling process, allowing us to use this information to improve the reconstruction. By reconstructing iteratively from smaller to larger image scales, we utilize our knowledge about the smaller scale image until the final scale is reconstructed (see Fig. 8). Our reconstruction method utilizes this idea and improves the reconstruction quality at a slight cost of running time.

## Supplementary Information

Supplementary Information.

## Data Availability

The datasets used and/or analyzed during the current study available from the corresponding author on reasonable request.
