# Supplementary material for: Progressive compressive sensing of large images with multiscale deep learning reconstruction
Source: Sci Rep. 2022 May 4;12:7228. doi: 10.1038/s41598-022-11401-7 (PMC9068919; doi:10.1038/s41598-022-11401-7)
Supplement: Supplementary file 1 — Supplementary Information. [file 41598_2022_11401_MOESM1_ESM.pdf]

Supplementary Material: Progressive Compressive Sensing of Large Images with Multiscale Deep Learning Reconstruction

Vladislav Kravets [*] and Adrian Stern

Ben-Gurion University of the Negev, Department of Electro-Optics and Photonics Engineering, School of Electrical and Computer Engineering, P.O.B. 653, Beer-Sheva 8410501, Israel
[*] kravetsv@post.bgu.ac.il

A. MULTISCALE SENSING SCHEME

In this section we prove eq. (3). First, we shall provide an intuition why the Paley ordered Hadamard matrix in eq. (2) exhibits a multiscale property we present in Fig. A1. the process of constructing the third order Hadamard matrix R_3. In the illustration, it can be seen that the lower ordered Hadamard matrix is repeated in the upper half of the higher ordered Hadamard matrix. This hints that the upper part of the signal vector sampled with the Hadamard matrix is the down-sampled signal sampled with a lower ordered Hadamard matrix.

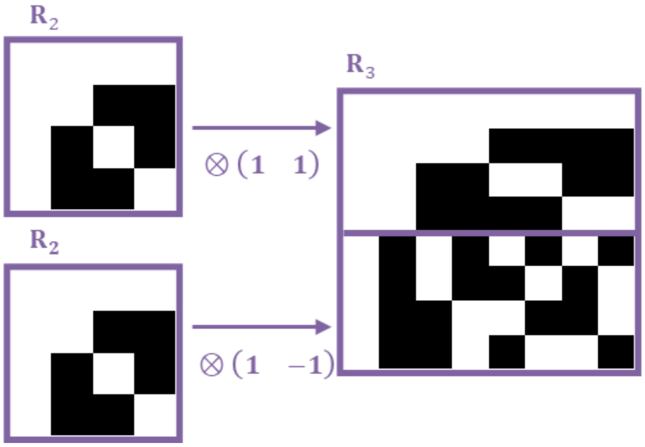

Fig. A1. The Paley ordered Hadamard matrix can be constructed from two lower level Paley ordered Hadamard matrices; demonstration on the Hadamard matrix $\boldsymbol{R}_{n=3}$ of size $2^3\times2^3$. The upper half of the matrix represents the process of decimation, while the lower half represents the high-frequency detail extraction.

One-Dimensional Multiscale Hadamard Transform

Paley ordered Hadamard transformation of the signal $\mathbf{f}\in\mathbb{R}^{2^n}$ can be defined by the multiplication of $\mathbf{f}$ by the matrix $\mathbf{R}\in\mathbb{R}^{2^n\times2^n}$ can be expressed in the following linear set of equations:

$$\mathbf{g}=\mathbf{R}_n\mathbf{f} \tag{A1}$$

where $\mathbf{R}$ is defined recursively by (2).

To show the multiscale property of the Hadamard transform we will explore the Hadamard-Haar relations. The Haar wavelet transform operation can be defined recursively with the help of the unitary matrix [1]:

$$\mathbf{W}_n=\frac{1}{\sqrt{2}}\begin{pmatrix}\mathbf{W}_{n-1}\otimes(1 \quad 1)\\ \mathbf{I}_{n-1}\otimes(1 \quad -1)\end{pmatrix}, \tag{A2}$$

where $\mathbf{W}_0=1$, and $\mathbf{I}_n\in\mathbb{R}^{2^n\times2^n}$ is the identity matrix. Then, the Haar transform of the signal $\mathbf{f}\in\mathbb{R}^{2^n}$ is given by

$$\mathbf{s}_n=\mathbf{W}_n\mathbf{f}=\frac{1}{\sqrt{2}}\begin{pmatrix}\mathbf{s}_{n-1}\\ \mathbf{d}_{n-1}\end{pmatrix}=\frac{1}{\sqrt{2}}\begin{pmatrix}\mathbf{W}_{n-1}\mathbf{a}_{n-1}\\ \mathbf{d}_{n-1}\end{pmatrix}= \tag{A3}$$

$$\begin{pmatrix} 2^{-1}\mathbf{W}_{n-2}\mathbf{a}_{n-2} \\ 2^{-1}\mathbf{d}_{n-2} \\ 2^{-1/2}\mathbf{d}_{n-1} \end{pmatrix} = \ldots = \begin{pmatrix} 2^{-n/2}\mathbf{a_0} \\ 2^{-n/2}\mathbf{d_0} \\ 2^{-n-1/2}\mathbf{d_1} \\ \vdots \\ 2^{-1/2}\mathbf{d_{n-1}} \end{pmatrix},$$

where $\mathbf{a}_{n-1} \in \mathbb{R}^{2^{n-1}}$ and $\mathbf{d}_{n-1} \in \mathbb{R}^{2^{n-1}}$ are the $n-1$ order *approximation* (lower resolution) and the *details* parts of the Haar wavelet transform, respectively, defined by:

$$\mathbf{a}_{n-1} = \begin{pmatrix} f_1 \\ f_3 \\ f_5 \\ \vdots \\ f_{n-1} \end{pmatrix} + \begin{pmatrix} f_2 \\ f_4 \\ f_6 \\ \vdots \\ f_n \end{pmatrix}$$

$$\mathbf{d}_{n-1} = \begin{pmatrix} f_1 \\ f_3 \\ f_5 \\ \vdots \\ f_{n-1} \end{pmatrix} - \begin{pmatrix} f_2 \\ f_4 \\ f_6 \\ \vdots \\ f_n \end{pmatrix}. \tag{A4}$$

The wavelet transformation of $\mathbf{f}_4$ is illustrated in Fig. A2.

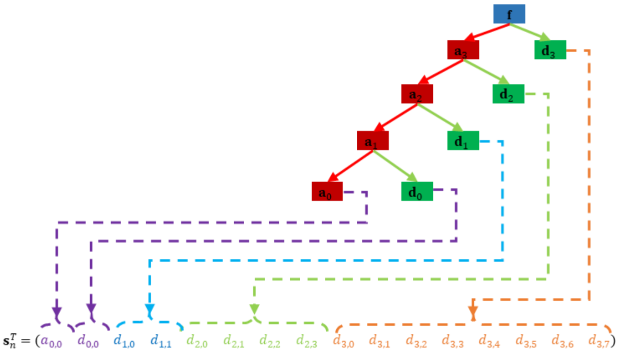

Fig. A2. Illustration of the wavelet transform of $\mathbf{f}_4$. The approximation (lower resolution) of the signal is depicted in red, while the detail of the signal is depicted in green. Notice the multiscale property of the wavelet transform.

To show the multiscale property of the Hadamard transform, we prove that if we separate $\mathbf{g}$ obtained by (A1) into an upper half $\mathbf{u} \in \mathbb{R}^{2^{n-1}}$ and lower half $\mathbf{v} \in \mathbb{R}^{2^{n-1}}$, so that:

$$\mathbf{g} = \begin{pmatrix} \mathbf{u} \\ \mathbf{v} \end{pmatrix}, \tag{A5}$$

we get:

$$\mathbf{u} = \frac{1}{\sqrt{2}} \mathbf{R}_{n-1} \mathbf{a}_{n-1}. \tag{A6}$$

Because $\mathbf{a}_{n-1}$ is the approximation (lower resolution) of the signal $\mathbf{f}$, it means that the upper half of the sample vector $\mathbf{g}$ is proportional to the Hadamard transform of the lower resolution level of $\mathbf{f}$. Furthermore, the same treatment (because the form of the equation is the same as Eq. (A1)) can be applied also on $\mathbf{u}$, meaning that the upper half of the vector $\mathbf{u}$ is proportional to the Hadamard transform of the lower resolution of $\mathbf{a}_{n-1}$, and so on. Thus (A6) defines a recursive relation between the Hadamard measurements to the multiscale approximation levels.

To prove (A6) we first prove that:

$$\mathbf{R}_n \cdot \mathbf{W}_n^T = 2 \begin{pmatrix} \mathbf{R}_{n-1}\mathbf{W}_{n-1}^T & \mathbf{0}_{n-1} \\ \mathbf{0}_{n-1} & \mathbf{R}_{n-1} \end{pmatrix}. \tag{A7}$$

Proof:

$$\mathbf{R}_n\cdot\mathbf{W}_n{}^{T}=\frac{1}{\sqrt{2}}\begin{pmatrix}\mathbf{R}_{n-1}\otimes(1\quad 1)\\ \mathbf{R}_{n-1}\otimes(1\quad -1)\end{pmatrix}\cdot$$
$$\cdot\left(\mathbf{W}_{n-1}^{T}\otimes\begin{pmatrix}1\\1\end{pmatrix}\quad \mathbf{I}_{n-1}\otimes\begin{pmatrix}1\\-1\end{pmatrix}\right). \tag{A8}$$

By using the Kronecker product relation [2] (A⊗B)(C⊗D)=(AC)⊗(BD):

$$\mathbf{R}_n\cdot\mathbf{W}_n{}^{T}=\frac{1}{\sqrt{2}}\begin{pmatrix}2\cdot\mathbf{R}_{n-1}\mathbf{W}_{n-1}^{T} & \mathbf{0}_{n-1}\\ \mathbf{0}_{n-1} & 2\cdot\mathbf{R}_{n-1}\end{pmatrix}=$$
$$=\sqrt{2}\begin{pmatrix}\mathbf{R}_{n-1}\mathbf{W}_{n-1}^{T} & \mathbf{0}_{n-1}\\ \mathbf{0}_{n-1} & \mathbf{R}_{n-1}\end{pmatrix}. \tag{A9}$$

Because $\mathbf{W_n}$ is unitary, we can write

$$\mathbf{g}=\mathbf{R}_n\mathbf{W}_n{}^{T}\mathbf{s}=\begin{pmatrix}\mathbf{R}_{n-1}\mathbf{W}_{n-1}^{T} & \mathbf{0}_{n-1}\\ \mathbf{0}_{n-1} & \mathbf{R}_{n-1}\end{pmatrix}\begin{pmatrix}\mathbf{s}_{n-1}\\ \mathbf{d}_{n-1}\end{pmatrix}. \tag{A10}$$

After separating $\mathbf{g}$ into an upper and lower half, we get:

$$\mathbf{u}=\mathbf{R}_{n-1}\mathbf{W}_{n-1}^{T}\mathbf{s}_{n-1}, \tag{A11}$$

and because $\mathbf{s}_{n-1}=\mathbf{W}_{n-1}\mathbf{a}_{n-1}$, we get (A6) as intended.

Two-Dimensional Multiscale Hadamard Transform

In this subsection, we generalize the property (A6) to two dimensional signals (images). The two-dimensional Hadamard transform of an image $\mathbf{F}\in\mathbb{R}^{2^n\times 2^n}$ can be written as [1]:

$$\mathbf{G}=\mathbf{R}_n\mathbf{F}\mathbf{R}_n, \tag{A12}$$

To show the multiscale property of the Hadamard transform in two dimensions, we will use Eq. (A6). First, we write

$$\mathbf{F}=(\mathbf{f}_1\quad \mathbf{f}_2\quad \cdots\quad \mathbf{f}_{2^n})$$
$$\mathbf{G}=\begin{pmatrix}\mathbf{u}_1 & \mathbf{u}_2 & \cdots & \mathbf{u}_{2^n}\\ \mathbf{v}_1 & \mathbf{v}_2 & \cdots & \mathbf{v}_{2^n}\end{pmatrix}, \tag{A13}$$

where $\mathbf{f}\in\mathbb{R}^{2^n}$ are the column vectors of $\mathbf{F}$, $\mathbf{u}\in\mathbb{R}^{2^{n-1}}$ are the upper half of $\mathbf{G}$ matrix column vectors and $\mathbf{v}\in\mathbb{R}^{2^{n-1}}$ are the lower half.

By applying the one-dimensional wavelet transform on the image columns and using Eq. (A6) we get:

$$(\mathbf{u}_1\quad \mathbf{u}_2\quad \cdots\quad \mathbf{u}_{2^n})=$$
$$=\frac{1}{\sqrt{2}}\mathbf{R}_{n-1}(\mathbf{a}_{n,1}\quad \mathbf{a}_{n,2}\quad \cdots\quad \mathbf{a}_{n,2^n})\mathbf{R}_n. \tag{A14}$$

Equation (A14) means that the upper part of the matrix $\mathbf{G}$ represents the Hadamard transform of the approximation (lower resolution) along the columns of the image $\mathbf{F}$ (see Fig. A3).

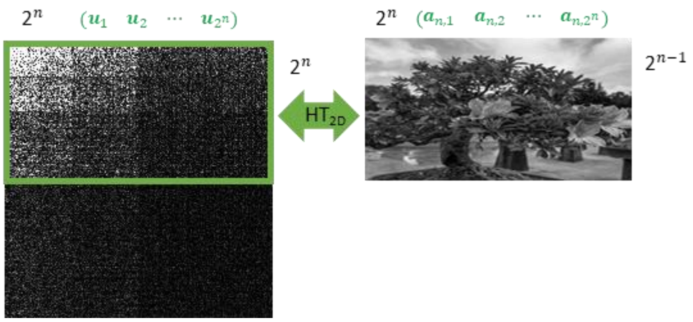

Fig. A3. Illustration of the result in Eq. (A14). When we take the upper half of the 2D Hadamard transform of the image (left), its inverse 2D Hadamard transform results in a down-sampled image along the vertical axes (right). Since the Hadamard transform is a unitary operator[1], the 2D Hadamard transform, and its inverse are both denoted by HT_{2D}.

Let us look at the transpose of eq. (A14) below

$$\begin{pmatrix} \mathbf{u}_1 \\ \mathbf{u}_2 \\ \vdots \\ \mathbf{u}_{2^{n-1}} \\ \mathbf{u}_{1+2^{n-1}} \\ \mathbf{u}_{2+2^{n-1}} \\ \vdots \\ \mathbf{u}_{2^n} \end{pmatrix} = \frac{1}{\sqrt{2}} \mathbf{R}_n \begin{pmatrix} \mathbf{a}_{n,1} \\ \mathbf{a}_{n,2} \\ \vdots \\ \mathbf{a}_{n,2^{n-1}} \\ \mathbf{a}_{n,1+2^{n-1}} \\ \mathbf{a}_{n,2+2^{n-1}} \\ \vdots \\ \mathbf{a}_{n,2^n} \end{pmatrix} \mathbf{R}_{n-1}. \tag{A15}$$

Repeating the one-dimensional wavelet transform again as in Eq. (A14), we get (See Fig. A4)

$$\mathbf{U}_{n-1}^{T} \equiv \begin{pmatrix} \mathbf{u}_1 \\ \mathbf{u}_2 \\ \vdots \\ \mathbf{u}_{2^{n-1}} \end{pmatrix} = \frac{1}{2} \mathbf{R}_{n-1} \mathbf{A}_{n-1}^{T} \mathbf{R}_{n-1}, \tag{A16}$$

where $\mathbf{A}_{n-1} \in \mathbb{R}^{2^{n-1} \times 2^{n-1}}$ is the approximation along the image $\mathbf{F}$ columns and then along its rows. We denote $\mathbf{U}_{n-1} \in \mathbb{R}^{2^{n-1} \times 2^{n-1}}$ as the upper left quarter of the sample matrix $\mathbf{G}$.

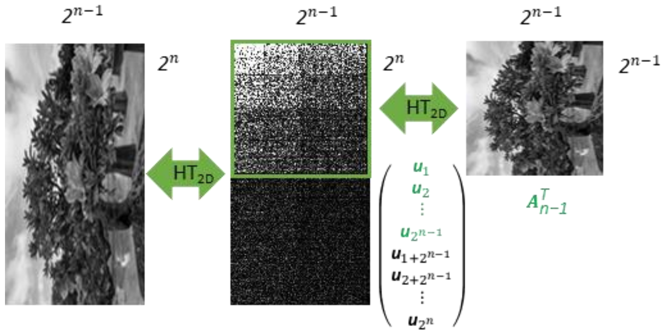

Fig. A4. Similar to the result in Eq. (A14), the upper (transposed) part of the Hadamard transform of the down-sampled image along the vertical axes is, in fact, the transpose of the down-sampled image on both axes. Since the Hadamard transform is a unitary operator[1], the 2D Hadamard transform, and its inverse are both denoted by HT_{2D}.

B. DEEP LEARNING NETWORK ARCHITECTURE

The proposed deep Convolutional Neural Network (CNN) architecture is illustrated schematically in Fig. B1 (a). Due to the way we reconstruct the compressed image, we can only use CNN frameworks that operate by pre-upsampling[3] the low-resolution image. Therefore, for our application, we considered networks such as the VDSR[4] and DRRN[5], and network design strategies introduced in ResNet[6], SRDenseNet[7] and RDN[8].

Inspired by the VDSR[4], the proposed Compressive Multi-Scale network (CMSnet) receives an approximation of the image and returns the predicted residual between the estimation and the original image. However, instead of using standard convolutional layers (as done with VDSR), we use five concatenation blocks (CB) illustrated schematically in Fig. B1 (b).

In a similar way to the ResNet[6], and SRDenseNet[7], we add a skip connection to each CB, followed by a depth concatenation instead of addition. As in the previous frameworks, we add a rectified linear unit (ReLU) after each convolutional layer. The purpose of the skip connection is to propagate a coarse approximation and to mitigate vanishing gradients[3, 9]. After the concatenation, we add an additional convolutional layer. The purpose of this layer is to compress the number of features by the factor of two, de facto forcing the network to use a weighted average of the features after the skip connection, instead of addition.

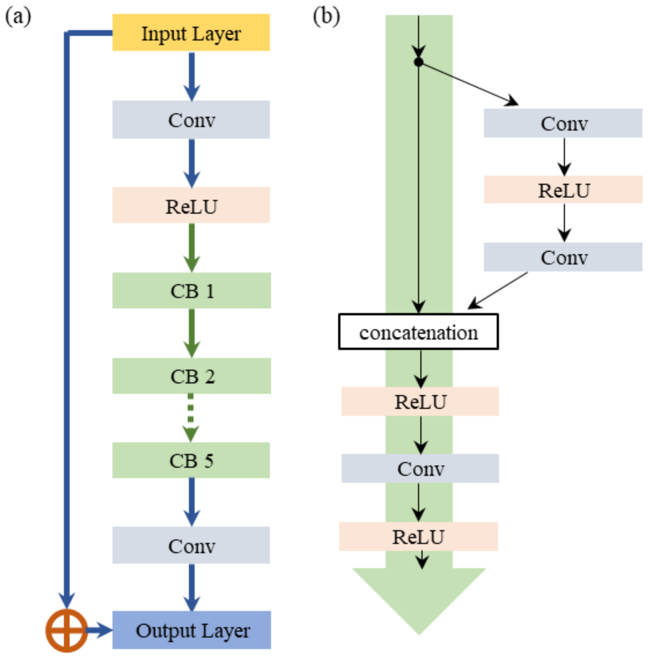

Fig. B1. The proposed CNN (a) is constructed from five concatenation blocks (CB) (b) (see Tables B1 ad B2 for lock sizes).

We summarize CMSnet's layers in **Table B1** and CB layers in **Table B2**. For each layer, we present the dimension of feature maps at the output (activations), and the dimensions of the weights and bias of the convolutional layers.

Type	Activations	Weights	Bias
Input	41x41x1	-	-
Conv	41x41x64	3x3x1x64	1x1x64
ReLU	41x41x64	-	-
CB1	41x41x64	-	-
⋮	⋮	⋮	⋮
CB5	41x41x64	-	-
Conv	41x41x1	3x3x64	1x1
Regression Outp.	-	-	-

Table B1. CMSnet architecture.

Type	Activations	Weights	Bias
Conv	41x41x64	3x3x64x64	1x1x64
ReLU	41x41x64	-	-
Conv	41x41x64	3x3x64x64	1x1x64
Concat.	41x41x128	-	-
ReLU	41x41x128	-	-
Conv	41x41x64	3x3x128x64	1x1x64
ReLU	41x41x64	-	-

Table B2. Concatenation Block architecture.

The proposed CMSnet was trained on a combination of 3 image datasets - DiV2K[10] which contains 800 images, Flickr2K[11] which contains 2650 images and Outdoor Scene Training[12] which contains over 10000 images. Each image was converted to

grayscale representation and cropped to an n-by-n resolution. We trained a different network for each range of compression ratios, in jumps of 10 (e.g., M/N = 0.1-0.2, 0.2-0.3, …). The test and validation images consisted of images separate from the database.

One of the most important aspects of the proposed multiscale sensing scheme is the variable density sampling. In contrast to the random compressive sampling with the Hadamard patterns, the variable density sampling makes the inverse Hadamard transform of the compressed samples closely resemble the original uncompressed image. This key aspect makes it possible to apply a convolutional neural network directly to this first approximation of the image and therefore, it can work on any image of any size. This is in contrast with alternative compressive sampling reconstruction methods that would have to apply a fully connected layer in order to build this first approximation layer. Such fully connected layer will be prohibitively expensive computationally, due to the high amount of weights needed to be optimized.

To demonstrate this aspect, in Fig. B2 we show what the reconstruction of a high-resolution image with the CNN method, would look like after sampling the scene with random Hadamard patterns. We reconstruct the image with the same proposed CNN but trained on the random Hadamard patterns. We compare the reconstruction result with our sensing and reconstruction approach, trained on the same CNN but utilizing the multiscale property of the Hadamard sampling matrix. Notice that the scene was sampled by the same amount of the same Hadamard samples. The only difference is that while during the naïve approach we choose the samples uniformly at random, with the proposed approach, we chose the samples at random but with variable density, preferring more lower sequency samples rather than samples at higher sequency.

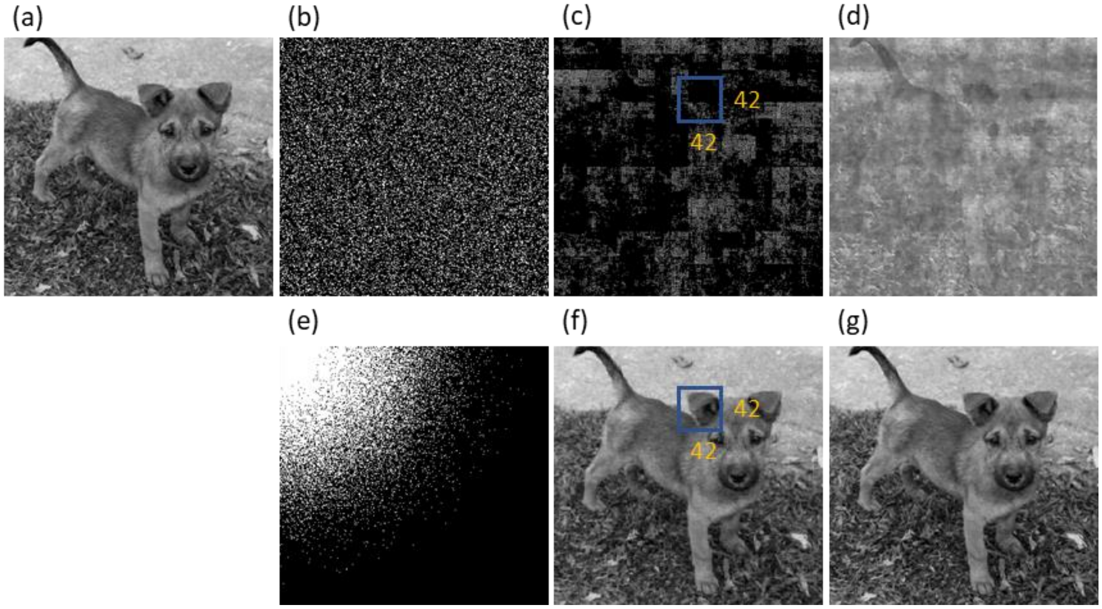

Fig. B2. (a) Original image. (b) Sampling map of the Hadamard samples ordered by their sequency (which is equivalent to frequency in Fourier transform) in 2D[1]. Samples that are taken (20%) are depicted in white, all the sequencies have the same chance to be selected for the sampling. (c) The inverse Hadamard transform of the samples, taken according to (b). (d) The image, reconstructed from (c) by the proposed CNN (PSNR = 13.14dB, SSIM = 0.45). Note that while the reconstruction is performed on the whole image, the network was trained on small 42 by 42 patches (example in the blue rectangle in (c)) and therefore any low frequency artifact larger than the sample will not be solved during the reconstruction. (e) Sampling map of the Hadamard samples ordered by their sequency in 2D. Samples that are taken (20%) are depicted in white, samples with low sequency (starting from the top-left corner) have a higher chance to be selected over the high sequency samples. (f) The inverse Hadamard transform of the samples, taken according to (e). (g) The image reconstructed from (f) by the proposed CNN (PSNR = 31.55dB, SSIM = 0.88).

From Fig. B2 it is apparent that it is very difficult to reconstruct an image with a CNN, after taking random Hadamard samples, contrary to iterative, non-CNN methods, which might reconstruct the image in such a case. This extremely poor reconstruction result can be explained by the fact that there are many very low-resolution samples missing during the compressive sampling process. This can be seen in Fig. B2 (c), the inverse Hadamard transform of the random Hadamard samples is missing a lot of low frequency and DC information, resulting in large block artifacts. Because the network is only trained on small 42 by 42 patches, it is impossible for it to reconstruct an image that has patterns of a larger size. On the

contrary, the inverse Hadamard transform of the image, sampled after the proposed multiscale approach (Fig. B2(f)), has all the required DC and low frequency information, thus making it possible to use a convolutional network that was trained on small patches.

The most straightforward way to apply the CMSnet on the compressed multiscale Hadamard samples is to apply it directly to the Hadamard transform of the compressed samples as shown in Fig. B3.

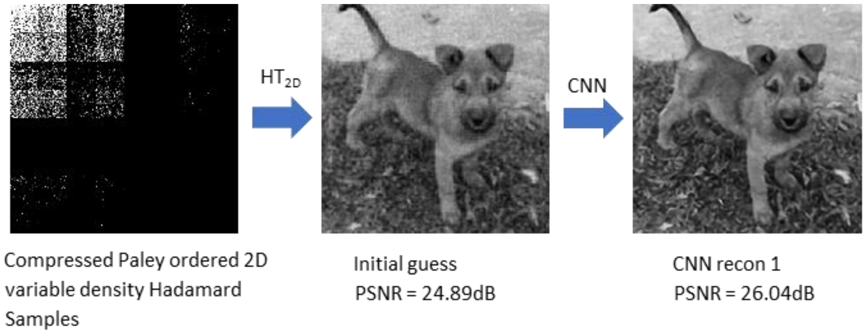

Fig. B3. Reconstruction of the compressed multiscale Hadamard samples directly by the CMSnet.

To improve the reconstruction quality, it is also possible to apply the reconstruction iteratively. We can add the compressive samples to the newly reconstructed image and apply the CMSnet again to improve the quality (see Fig. B4). This process can be repeated multiple times until there is no further improvement to the reconstruction quality. In our research, we found that after 3 times, the image quality no longer improves.

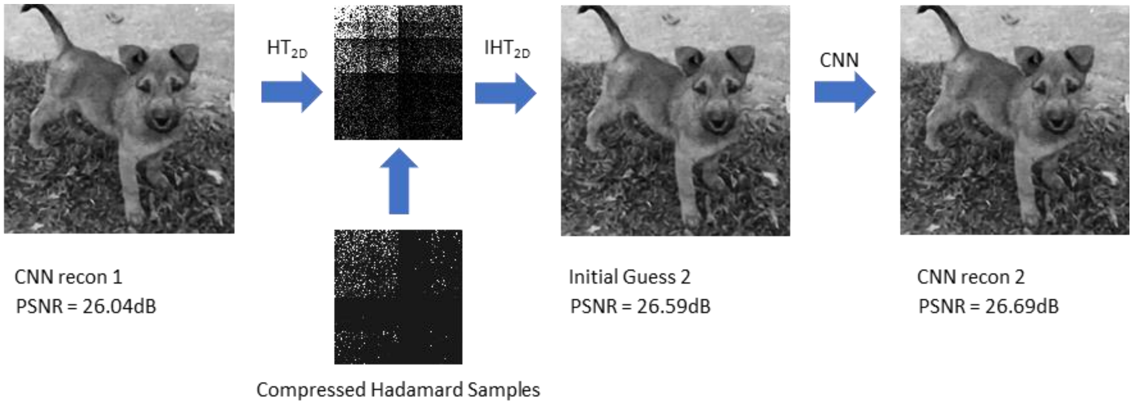

Fig. B4. To improve the reconstruction quality the network can be applied iteratively. We propose to add the original compressed samples to the reconstructed image and apply the CMSnet again to improve the reconstruction.

This reconstruction method works well, however, the reconstruction quality can be further improved by using the multiscale information about the image that was obtained during the sampling process. We reconstruct each scale of the image from low to high resolution. A heuristic way to show how this multiscale information helps in reconstruction, is to look at the sampling patterns of the Hadamard matrix (see Fig. B5). In Fig. B5 we start from the initial sampling map of the 2D Hadamard transform, ordered sequentially. The samples taken are depicted in beige color and the missing samples are depicted in black. By using the multiscale property of the Hadamard transform, we estimate the 128 by 128 image from the samples by using the proposed CNN. We then can fill in the missing, black samples by taking the Hadamard transform of the estimated image. The process is repeated iteratively until the final resolution is achieved.

Notice that in the final step, only the high sequency samples are missing while all the low sequency points are already filled in by the estimation from the previous step. This way there are no low frequency patterns that are larger than the 42 by 42 patches that the network was trained on (and therefore couldn't reconstruct) and we can prevent the reconstruction artifacts depicted in Fig. B2 (d).

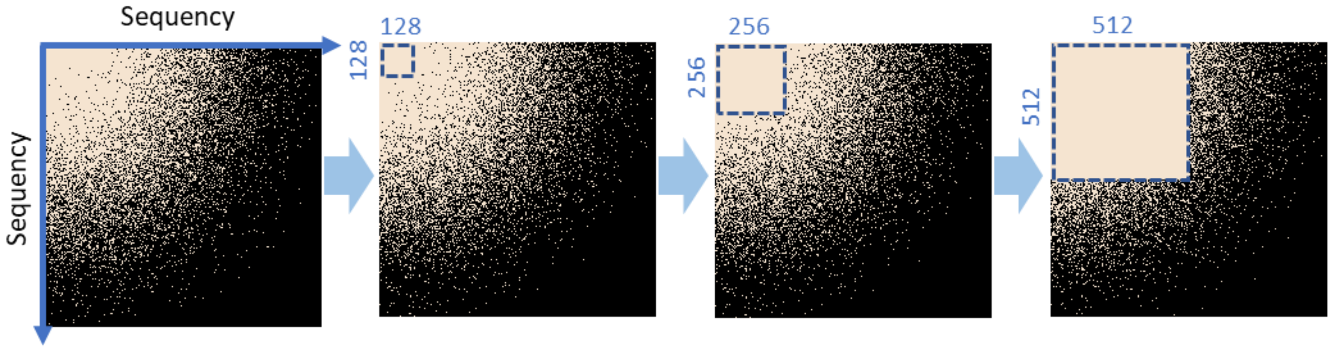

Fig. B5. Sampling map of the Hadamard samples ordered by their sequency in 2D. Points depicted in beige color are the non-zero samples of the scene, sampled by the Hadamard patterns. By using the multiscale property of the Hadamard basis a 128 by 128 low resolution image is reconstructed and the missing points in the sampling map can be filled in by the approximation. Then, the 256 by 256 image is reconstructed from the new samples and a new sampling map is estimated by filling up the missing samples with the new estimation.

The complete reconstruction procedure is performed in two steps. In the first step, we generate the first estimation of the image, and in the second step, we plug the estimation into the proposed CNN in order to receive the final image.

At the first stage of the progressive sampling, where the first resolution level is captured and, hence, no information of a smaller resolution image is available, the first estimation of the image is taken as the inverse Hadamard transform of the compressed Hadamard samples. In the following stages of the progressive sampling, the already available lower resolution reconstruction is up-sampled by a Bicubic interpolation. After the interpolation, the image is converted to the Paley ordered Hadamard domain and its entries are substituted with the additional compressive Hadamard samples, ΔM. We then restore the image back to the spatial domain, but this time, including the newly measured compressive Hadamard samples. This approximation process is illustrated in Fig. B6.

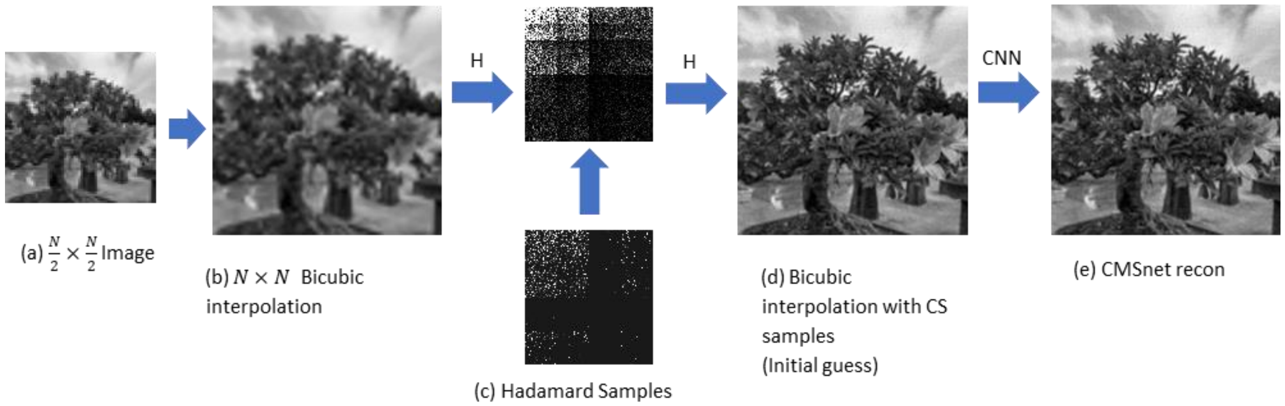

Fig. B6. The process of adding the compressive samples ΔM to the lower resolution image. A lower resolution image (a) is upscaled using the Bicubic interpolation. The approximation of the image (b) is transformed to the Hadamard basis, after which the newly measured compressive Hadamard samples are used to substitute the points of the Bicubic approximation at the Hadamard basis (c). Then, the image is converted back by using the Hadamard transform (d) and this approximation is used as an input to the CMSnet for the final reconstruction (e).

In the second step, we plug the obtained image into the CMSnet designed for this purpose. Finally, we reconstruct the image by adding the predicted residual to the estimated image. In order to improve the reconstruction quality, the second step can be applied iteratively (See Fig. B4).

A typical multiscale sampling procedure would require the user to prepare beforehand the variable sampling strategy. For example, let's imagine that it was decided to use M/N = 0.125 compression at a 256x256 scale. Following variable density sampling [13], this would mean that at 64x64 scale, M/N = 0.91, and at 128x128 scale, M/N = 0.48. Now, we assume that a human observer is in the loop, sampling the scene at its lowest resolution, 64x64. If the observer cannot identify the target at its lowest resolution, he may request additional samples, predetermined by the sampling strategy. This would mean that

to achieve the M/N = 0.48 ratio at 128x128, only M/N = 0.26 samples would be added. If the quality is still unsatisfactory, the observer can request additional M/N = 0.004 samples to achieve M/N = 0.10 compression ratio at 256x256 scale. Following this scenario, only a single scale would need to be reconstructed by the CMSnet each time.

To show the effectiveness of the proposed multiscale sampling and reconstruction approach, we compare our reconstruction method with other DL compressive reconstruction methods in tables B3 and B4. The methods chosen for the first comparison are ReconNet[14], ISTA-Net[15] and CSNet[16]. The comparison was run on Set11[14] containing eleven 256 by 256 grayscale images. We can see that our method is superior for this kind of application in terms of PSNR over other methods in table B3.

For the second comparison, we compare our method to CSGAN[17], ReconNet[14] and SCGAN[18] trained on cropped 64 by 64 images of faces from the CelabA[19] dataset. For testing, we have used 20,000 face images from the test set. We can see that our method is superior for this kind of application in terms of PSNR over other methods in table B4.

For the third comparison, we compared our multiscale sensing and reconstruction method with two adaptive sampling methods[20, 21]. Our method achieved 31.75dB reconstruction PSNR from $M = 12{,}796$ compressive samples of the "*Lena*" image, while ADS[20] achieved 28.7dB PSNR under the same conditions. On the "*house*" image, after simulating shot-noise and detector read-out noise, our method achieved 29.31dB SNR at M/N = 6.25% compression, while the wavelet three parsing method[21] achieved 22.8dB SNR.

Algorithm	Sampling Ratio					
	0.5	0.4	0.3	0.1	0.01	Avg.
ReconNet	31.50	30.58	28.74	24.28	17.27	26.474
ISTA-Net+	38.07	36.06	33.82	26.64	17.34	30.386
CSNet+	38.52	36.48	34.30	28.37	21.03	31.74
CMSnet	**39.98**	**37.86**	**35.05**	**29.13**	**21.65**	**32.734**

Table B3. PSNR comparison between the proposed multiscale sampling and reconstruction method, and alternative DL reconstruction methods on Set11 test images. Best PSNR is highlighted in bold.

No. of measurements	CSGAN		ReconNet		SCGAN		CMSnet	
	PSNR	SSIM	PSNR	SSIM	PSNR	SSIM	PSNR	SSIM
20	13.95	0.17	18.39	0.32	20.21	0.44	**20.62**	**0.50**
50	18.50	0.36	19.24	0.37	**22.59**	0.56	22.52	**0.61**
100	20.25	0.46	22.05	0.52	24.47	0.65	**24.75**	**0.73**
200	22.00	0.54	24.24	0.64	26.42	0.73	**27.33**	**0.85**
500	22.26	0.54	26.77	0.75	28.40	0.80	**31.73**	**0.94**

Table B4. PSNR and SSIM comparison between the proposed multiscale sampling and reconstruction method, and alternative DL reconstruction methods on CelebA dataset. Best PSNR and SSIM is highlighted in bold.

C. SPC experiment results

In figure C1 below we presented experimental results, taken with the multiscale CS SPC imager, and reconstructed by CMCnet from only $M/N = 1\%$ compressive samples.

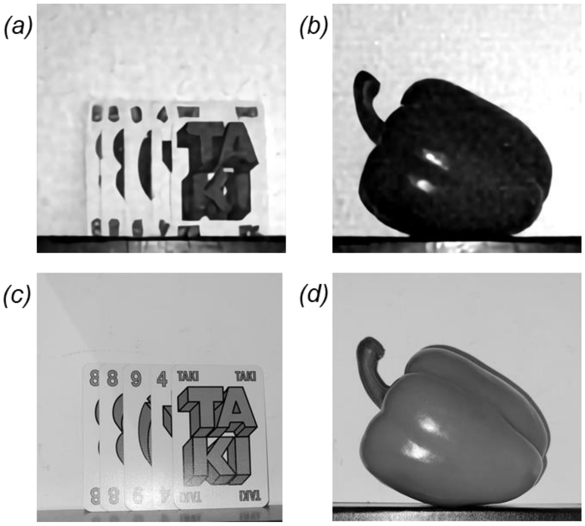

Fig C1. (a-b) Real 512x512 reconstructed images at M/N = 1%, captured by an SPC and reconstructed by the CMSnet. (c-d) Reference images, taken by a high-resolution camera.

References

1. Sos S. Agaian, Hakob G. Sarukhanyan, Karen O. Egiazarian & Jaakko Astola. in *Hadamard transforms* 1-13 (SPIE Press, Bellingham, Wash, 2011).
2. Jain, A. K. in *Fundamentals of digital image processing* 30-31 (Englewood Cliffs, NJ: Prentice Hall, 1989).
3. Wang, Z., Chen, J. & Hoi, S. C. Deep learning for image super-resolution: A survey. *IEEE Trans. Pattern Anal. Mach. Intell.* (2020).
4. Kim, J., Kwon Lee, J. & Mu Lee, K. *Accurate image super-resolution using very deep convolutional networks* (Proceedings of the IEEE conference on computer vision and pattern recognition, 2016).
5. Tai, Y., Yang, J. & Liu, X. *Image super-resolution via deep recursive residual network* (Proceedings of the IEEE conference on computer vision and pattern recognition, 2017).
6. Huang, G., Liu, Z., Van Der Maaten, L. & Weinberger, K. Q. *Densely connected convolutional networks* (Proceedings of the IEEE conference on computer vision and pattern recognition, 2017).
7. Tong, T., Li, G., Liu, X. & Gao, Q. *Image super-resolution using dense skip connections* (Proceedings of the IEEE International Conference on Computer Vision, 2017).
8. Zhang, Y., Tian, Y., Kong, Y., Zhong, B. & Fu, Y. *Residual dense network for image super-resolution* (Proceedings of the IEEE Conference on Computer Vision and Pattern Recognition, 2018).
9. He, K., Zhang, X., Ren, S. & Sun, J. *Deep residual learning for image recognition* (Proceedings of the IEEE conference on computer vision and pattern recognition, 2016).
10. Agustsson, E. &Timofte, R. *Ntire 2017 challenge on single image super-resolution: Dataset and study* (Proceedings of the IEEE conference on computer vision and pattern recognition workshops, 2017).
11. Timofte, R., Agustsson, E., Van Gool, L., Yang, M. & Zhang, L. *Ntire 2017 challenge on single image super-resolution: Methods and results* (Proceedings of the IEEE conference on computer vision and pattern recognition workshops, 2017).
12. Wang, X., Yu, K., Dong, C. & Loy, C. C. *Recovering realistic texture in image super-resolution by deep spatial feature transform* (Proceedings of the IEEE conference on computer vision and pattern recognition, 2018).
13. Wang, Z. & Arce, G. R. Variable Density Compressed Image Sampling. *IEEE Transactions on Image Processing* **19**, 264-270 (2010).
14. Kulkarni, K., Lohit, S., Turaga, P., Kerviche, R. & Ashok, A. *Reconnet: Non-iterative reconstruction of images from compressively sensed measurements* (Proceedings of the IEEE Conference on Computer Vision and Pattern Recognition, 2016).
15. Zhang, J. &Ghanem, B. *ISTA-Net: Interpretable optimization-inspired deep network for image compressive sensing* (Proceedings of the IEEE conference on computer vision and pattern recognition, 2018).
16. Shi, W., Jiang, F., Liu, S. & Zhao, D. Image compressed sensing using convolutional neural network. *IEEE Trans. Image Process.* **29**, 375-388 (2019).

17. Kabkab, M., Samangouei, P. & Chellappa, R. *Task-aware compressed sensing with generative adversarial networks* (Proceedings of the AAAI Conference on Artificial Intelligence Ser. 32, 2018).
18. Sun, Y., Chen, J., Liu, Q. & Liu, G. Learning image compressed sensing with sub-pixel convolutional generative adversarial network. *Pattern Recognit* **98**, 107051 (2020).
19. Liu, Z., Luo, P., Wang, X. & Tang, X. Large-scale celebfaces attributes (celeba) dataset. *Retrieved August* **15**, 11 (2018).
20. Deutsch, S., Averbush, A. & Dekel, S. *Adaptive compressed image sensing based on wavelet modeling and direct sampling* (SAMPTA'09, 2009).
21. Saragadam, V. &Sankaranarayanan, A. C. *Wavelet tree parsing with freeform lensing* (2019 IEEE International Conference on Computational Photography (ICCP), IEEE, 2019).
